# A systematic review and meta-analysis of acoustic stimulation in the treatment of insomnia

**DOI:** 10.3389/fnins.2025.1572086

**Published:** 2025-06-13

**Authors:** Mengchen Wang, Shuai Fan, Zixu Wang, Jixiang Ren

**Affiliations:** ^1^Changchun University of Chinese Medicine, Changchun, China; ^2^Affiliated Hospital to Changchun University of Chinese Medicine, Changchun, China

**Keywords:** acoustic stimulation, insomnia treatment, sleep quality, non-pharmacological therapy, meta-analysis

## Abstract

**Background:**

Insomnia is a prevalent and debilitating sleep disorder affecting approximately one-third of the global population, with 10–15% of individuals progressing to chronic insomnia. Acoustic stimulation, a promising non-pharmacological treatment, has gained significant attention due to its ability to improve sleep quality without the adverse effects of drugs. This study aims to evaluate the clinical efficacy of acoustic stimulation for treating insomnia through a systematic review and meta-analysis.

**Methods:**

We conducted a systematic search of both Chinese and English literature from PubMed, Embase, Web of Science, Cochrane, and Clinical Trials databases to identify randomized controlled trials (RCTs) evaluating acoustic stimulation for insomnia. The search period ranged from the establishment of the database to January 1, 2025. Two independent evaluators assessed study quality and extracted relevant data. Statistical analyses were conducted using RevMan 5.4 and Stata 18.0 software.

**Results:**

This meta-analysis includes 8 studies involving 419 patients. The results showed that acoustic stimulation significantly improved insomnia severity as evidenced by PSQI and ISI scores. Despite no significant improvement in sleep efficiency (SE) and Total sleep time (TST), acoustic stimulation notably increased total sleep time and alleviated insomnia symptoms. The test group demonstrated significant improvements compared to the control group, with the following outcomes: PSQI score [MD = -2.68, 95% CI (-3.35, -2.01), *P* < 0.00001] and ISI score [MD = -2.26, 95% CI (-4.09, -0.43), *P* = 0.02].

**Conclusion:**

Acoustic stimulation is an effective and safe treatment for insomnia, offering significant improvements in sleep quality, severity, and overall health, with minimal side effects. It presents a promising alternative to traditional pharmacological treatments, especially for long-term use, and its clinical application has broad prospects. Future studies should focus on expanding sample sizes, exploring various stimulation methods, and considering individual patient characteristics for more detailed analyses.

**Systematic review registration:**

https://www.crd.york.ac.uk/PROSPERO/view/CRD42025632371, identifier CRD42025632371.

## 1 Introduction

Insomnia is one of the most common sleep disorders globally, affecting approximately one-third of the population at some point in their lives, with about 10–15% of individuals developing chronic insomnia (Morin and Buysse, 2024). Insomnia not only impairs sleep quality but also significantly affects daytime functioning, leading to mood instability, difficulty concentrating, and decreased work and study efficiency. Chronic insomnia is closely linked to various physical health problems, including hypertension, diabetes, and cardiovascular diseases, and can exacerbate mental health issues such as anxiety and depression (Javaheri and Redline, 2017). Therefore, the treatment of insomnia requires more than just improving sleep quality; it also necessitates a comprehensive approach that addresses the patient’s overall health. Furthermore, chronic insomnia has been associated with cognitive decline and an increased risk of developing dementia, including Alzheimer’s disease. It is also linked to autonomic nervous system dysfunction, characterized by elevated sympathetic nervous activity and reduced heart rate variability, both of which contribute to adverse cardiovascular and metabolic outcomes.

Traditional treatments for insomnia primarily rely on medications, such as benzodiazepines, antidepressants, and sedatives. While these drugs can provide short-term relief, prolonged use often results in drug dependency, increased drug resistance, and cognitive dysfunction, posing potential risks to both physical and mental health (Morin and Benca, 2012). As a result, there has been growing interest among both patients and healthcare professionals in non-pharmacological treatments, seeking safer, more sustainable alternatives with fewer side effects.

In recent years, acoustic stimulation has emerged as a promising non-drug therapy, gaining significant attention. Acoustic stimulation involves delivering sound waves of specific frequencies to the brain to regulate neural activity. It has been shown to improve mood, regulate the circadian rhythm, alleviate anxiety and stress, and promote deep sleep, significantly enhancing sleep quality. Unlike traditional drug therapies, acoustic stimulation does not rely on medication, avoiding the side effects associated with pharmacological treatments and helping patients regulate their physiological rhythms and neural functions, thereby improving both sleep and psychological wellbeing (Kanzler et al., 2023).

Although several studies have explored the potential of acoustic stimulation for insomnia treatment, the available literature is still limited, and the results of these studies show considerable heterogeneity. Therefore, this study aims to conduct a meta-analysis, synthesizing the existing clinical data to evaluate the overall effectiveness of acoustic stimulation in treating insomnia, thus providing more reliable evidence for its clinical application. Additionally, this paper will examine the specific effects of different types of acoustic stimulation in insomnia treatment, offering theoretical insights for future research and clinical practice.

## 2 Methods

### 2.1 Date source and search strategy

A search was conducted in PubMed, Embase, Web of Science, Cochrane, and Clinical Trials databases for all relevant literature on clinical randomized controlled trials of acoustic stimulation for the treatment of insomnia. The search period was set from the time of database establishment to January 1, 2025. A combination of subject and free word searches was used. The specific search formula is as follows: English search formula:(((“ulation, as a non-pharmacologicSleep Initiation and Maintenance Disorders”[Mesh]) OR (((((((((((((((((((((((((Disorders of Initiating [Title/Abstract] AND Maintaining Sleep[Title/Abstract]) OR (Sleeplessness[Title/Abstract])) OR (Insomnia Disorder [Title/Abstract])) OR (Insomnia Disorders[Title/Abstract])) OR (Insomnia[Title/Abstract])) OR (Insomnias[Title/Abstract])) OR (Chronic Insomnia[Title/Abstract])) OR (Insomnia, Chronic[Title/Abstract])) OR (Early Awakening[Title/Abstract])) OR (Awakening, Early[Title/Abstract])) OR (Non-organic Insomnia[Title/Abstract])) OR (Insomnia, Non-organic[Title/Abstract])) OR (Primary Insomnia[Title/Abstract])) OR (Insomnia, Primary[Title/Abstract])) OR (Psychophysiological Insomnia[Title/Abstract])) OR (Insomnia, Psychophysio- logical[Title/Abstract])) OR (Rebound Insomnia[Title/Abstract])) OR (Insomnia, Rebound[Title/Abstract])) OR (Secondary Insomnia[Title/Abstract])) OR (Insomnia, Secondary[Title/Abstract])) OR (Sleep Initiation Dysfunction[Title/Abstract])) OR (Dysfunction, Sleep Initiation[Title/Abstract])) OR (Dysfunctions, Sleep Initiation[Title/Abstract])) OR (Sleep Initiation Dysfunctions[Title/Abstract])) OR (Transient Insomnia[Title/Abstract]))) AND ((“Acoustic Stimulation”[Mesh]) OR (((((((((((((((((((((((((Stimulation, Auditory[Title/Abstract]) OR (Stimulation, Acoustic[Title/Abstract])) OR (Auditory Stimulation[Title/Abstract])) OR (Sound Stimulation [Title/Abstract])) OR (Auditory Exposure[Title/Abstract])) OR (Acoustic Therapy[Title/Abstract])) OR (Sound Therapy[Title/Abstract])) OR (Noise Therapy[Title/Abstract])) OR (White Noise[Title/Abstract])) OR (Pink Noise[Title/Abstract])) OR (Binaural Beats[Title/Abstract])) OR (Noise Masking[Title/Abstract])) OR (Sound Masking[Title/Abstract])) OR (Tinnitus Sound Therapy [Title/Abstract])) OR (Music Therapy[Title/Abstract])) OR (Sound-based Therapy[Title/Abstract])) OR (Music-Based Intervention[Title/Abstract])) OR (Low-Frequency Sound[Title/Abstract])) OR (High-Frequency Sound[Title/Abstract])) OR (Sonic Stimulation[Title/Abstract])) OR (Rhythmic Auditory Stimulation[Title/Abstract])) OR (Auditory Feedback[Title/Abstract])) OR (Vocal Stimulation[Title/Abstract])) OR (Environmental Sound Therapy[Title/Abstract])) OR (Musical Sound Stimulation[Title/Abstract])))) AND ((randomized controlled trial[pt] OR controlled clinical trial[pt] OR clinical trials as topic[mesh:noexp] OR trial[ti] OR random*[tiab] OR placebo*[tiab])).

### 2.2 Literature inclusion criteria

#### 2.2.1 Study type

Clinical randomized controlled trials, and the language of the literature was restricted to English.

#### 2.2.2 Study subjects

Adults diagnosed with insomnia based on clinical standards or validated sleep assessment tools (e.g., ISI, PSQI), with comparable baseline data; free from other comorbidities and capable of clearly expressing changes in their condition.

#### 2.2.3 Intervention study

The experimental group received acoustic stimulation therapy, while the control group participants either received no treatment or placebo interventions (e.g., sham acoustic interventions or neutral sounds). The study aimed to evaluate the effects of acoustic stimulation compared to non-active interventions. Participants in the experimental group were not allowed to take insomnia treatment medications prior to the intervention.

#### 2.2.4 Outcome indicators

The primary outcome measures included the Pittsburgh Sleep Quality Index (PSQI), total sleep time (TST), sleep efficiency (SE), and the Insomnia Severity Index (ISI). The PSQI evaluates subjective sleep quality over the past month, with scores ranging from 0 to 21; higher scores indicate poorer sleep quality, and a score above 8 generally suggests clinically significant sleep disturbance. The ISI assesses insomnia severity over the past 2 weeks, with a total score ranging from 0 to 28; scores of 8–14 indicate subthreshold insomnia, 15–21 indicate moderate insomnia, and 22–28 indicate severe insomnia.

TST and SE were obtained either from self-reported sleep diaries or, when available, from objective polysomnography (PSG) assessments. When both were reported, PSG-derived data were prioritized to enhance measurement accuracy. A decrease of ≥ 3 points in PSQI and ≥ 6 points in ISI is typically regarded as a clinically meaningful improvement.

#### 2.2.5 Exclusion criteria

• Reviews, conference abstracts, case reports, animal experiments, and case treatment literature;
• Duplicate publications from the same experiment;
• Studies where the experimental group and control group are inconsistent or lack comparability;
• Studies with incomplete data or data that cannot be pooled;
• Studies where acoustic stimulation is not the primary intervention or is combined with other non-sound-based therapies (e.g., pharmacological treatments);
• Studies employing non-auditory intervention methods, such as cognitive behavioral therapy or light therapy.

### 2.3 Data extraction and analysis

#### 2.3.1 Data extraction

Two researchers independently screened the literature in separate environments based on inclusion and exclusion criteria. Data extraction was conducted using an Excel-designed extraction form, which included the following: (1) basic information such as publication year, country of the study, first author, and baseline characteristics; (2) sample size, interventions, and duration of the study for both the experimental and control groups; (3) endpoint data from each included study; and (4) details related to the quality assessment of the literature. After completing the extraction, the two researchers exchanged and compared their results. In case of discrepancies, the issue was resolved through discussion between the researchers or, if necessary, by consulting a third-party expert to ensure the accuracy and consistency of the data extraction.

#### 2.3.2 Quality assessment

The quality of the literature was assessed using the Cochrane Collaboration’s built-in tool in RevMan 5.4, focusing on the following aspects: (1) implementation of randomization; (2) allocation concealment; (3) blinding of researchers and participants; (4) blinding of outcome assessors; (5) completeness of outcome data; (6) presence of selective reporting; and (7) other potential biases. The risk assessment results were categorized as high risk, low risk, or unclear risk.

#### 2.3.3 Statistical methods

Meta-analysis was conducted using RevMan 5.4 software. Continuous variable effect sizes were expressed as mean differences (MD) with 95% confidence intervals (CI), and statistical significance was set at *P* < 0.05. Heterogeneity among studies was evaluated based on the *I*^2^ value: when *P* ≥ 0.1 and *I*^2^ < 50%, heterogeneity was considered minimal or absent, and a fixed-effects model was used. Conversely, significant heterogeneity was addressed using a random-effects model, with further exploration of heterogeneity sources through sensitivity or subgroup analyses. When at least 10 studies were included, publication bias assessment was deemed meaningful and could be evaluated using a funnel plot or quantitative data provided by the Egger test.

## 3 Results

### 3.1 Description of the selected studies

A total of 500 articles were initially retrieved from English-language databases. After removing duplicates, 372 articles remained for title and abstract screening. Following this initial screening, 54 articles were excluded due to irrelevance to the research topic. An additional 16 articles were excluded after full-text review based on the following criteria: incomplete data (*n* = 2), outcome measures that did not meet the predefined inclusion criteria (*n* = 2), and control groups that did not satisfy eligibility requirements (*n* = 4).

**FIGURE 1:**
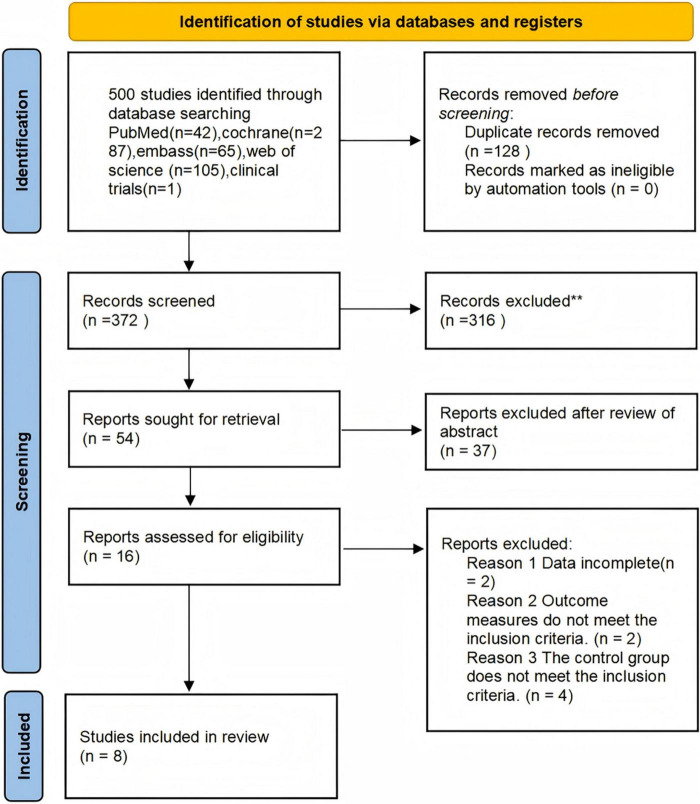
PRISMA flow diagram for article selection.

Specifically, the two studies categorized under “data incomplete” were excluded due to the absence of pre-post intervention comparisons or full-text availability. Ultimately, 8 studies were included in the meta-analysis. The study selection process is illustrated in Figure 1 (Chang et al., 2012; Dudysová et al., 2024; Harmat et al., 2008; Jespersen et al., 2019; Lai and Good, 2005; Tegeler et al., 2020; Tegeler et al., 2023; Wang et al., 2016).

### 3.2 Characteristics of included studies

A total of 8 studies included 419 patients, with 214 in the experimental group and 205 in the control group (Chang et al., 2012; Dudysová et al., 2024; Harmat et al., 2008; Jespersen et al., 2019; Lai and Good, 2005; Tegeler et al., 2020; Tegeler et al., 2023; Wang et al., 2016).

#### 3.2.1 Interventions

The experimental group received acoustic stimulation therapy. In the control group, 3 studies utilized closed-loop auditory stimulation (Dudysová et al., 2024; Tegeler et al., 2020; Tegeler et al., 2023), and 5 studies applied music therapy (Harmat et al., 2008; Jespersen et al., 2019; Lai and Good, 2005; Tegeler et al., 2023; Wang et al., 2016). The treatment duration ranged from 2 days to 6 weeks.

#### 3.2.2 Outcome measures

Five studies reported Pittsburgh Sleep Quality Index (PSQI) scores (Harmat et al., 2008; Jespersen et al., 2019; Lai and Good, 2005; Tegeler et al., 2023; Wang et al., 2016), three studies reported Insomnia Severity Index (ISI) scores (Jespersen et al., 2019; Tegeler et al., 2020; Tegeler et al., 2023), three studies provided total sleep time (TST) data (Chang et al., 2012; Dudysová et al., 2024; Jespersen et al., 2019), and three studies reported sleep efficiency (SE) (Chang et al., 2012; Dudysová et al., 2024; Jespersen et al., 2019). To clearly present differences among the included studies in terms of sample size, age, sex distribution, intervention type, and control condition, a summary table has been added (Table 1).

**TABLE 1:** Basic characteristics of the included studies.

	Sample size/case	Average age/year		Gender (sex, females/males, *n*)			Intervention	
**Inclusion of studies**	**T/C**	**T**	**C**	**T**	**C**	**Treatments**	**Test group**	**Control subjects**
Chang et al. (2012)	25/25	30.88 ± 10.89	32.76 ± 11.45	23/2	24/1	3 days	Music	No intervention
Dudysová et al. (2024)	7/7	28.0 ± 13.86	28.0 ± 13.86	3/4	3/4	2 days	Closed-loop auditory stimulation	Sham stimulus
Harmat et al. (2008)	35/29	22.6 ± 2.83	22.6 ± 2.83	NR	NR	3 weeks	Music group	No intervention
Jespersen et al. (2019)	19/19	50.9 ± 10.9	51.6 ± 8.2	15/4	15/4	3 weeks	Music	No intervention
Lai and Good (2005)	30/30	67 ± 5	67 ± 5	NR	NR	3 weeks	Music	No intervention
Tegeler et al. (2020)	56/51	52.4 ± 15.1	54.7 ± 14.8	41/15	32/19	3 weeks	HIRREM	Random tone
Tegeler et al. (2023)	10/12	58.6 ± 8.0	54.7 ± 19.2	7/3	9/3	6 weeks	Cereset research	Random tone
Wang et al. (2016)	32/32	66.94 ± 4.99	69.82 ± 5.61	26/8	29/5	12 weeks	Music	Sleep hygiene education

T, Treatment group; C, Control subjects; NR, not report.

### 3.3 Estimation of quality

All 8 included studies used standardized randomization methods, ensuring proper randomization (Chang et al., 2012; Dudysová et al., 2024; Harmat et al., 2008; Jespersen et al., 2019; Lai and Good, 2005; Tegeler et al., 2020; Tegeler et al., 2023; Wang et al., 2016). When assessed with the Cochrane Risk of Bias tool for random sequence generation, the risk was determined to be low.

All studies implemented allocation concealment methods, such as sealed envelopes or independent group assignments (Chang et al., 2012; Dudysová et al., 2024; Harmat et al., 2008; Jespersen et al., 2019; Lai and Good, 2005; Tegeler et al., 2020; Tegeler et al., 2023; Wang et al., 2016), effectively avoiding allocation bias, resulting in a low-risk assessment.

Regarding blinding, two studies employed sham interventions to achieve blinding, reducing implementation bias, and were assessed as low risk (Dudysová et al., 2024; Tegeler et al., 2023). Although full binding is inherently challenging in auditory stimulation studies, due to the perceptible nature of sound, these two studies employed randomly generated tones that were not linked to brainwave activity to create a sham condition, thereby partially achieving participant blinding and helping to mitigate performance bias. The remaining six studies, due to the nature of the intervention (e.g., music therapy or acoustic stimulation), could not blind participants and researchers, leading to a high-risk assessment (Chang et al., 2012; Harmat et al., 2008; Jespersen et al., 2019; Lai and Good, 2005; Tegeler et al., 2020; Wang et al., 2016).

For data integrity, six studies had complete data with all participants finishing the intervention, resulting in a low-risk assessment (Chang et al., 2012; Harmat et al., 2008; Lai and Good, 2005; Tegeler et al., 2020; Tegeler et al., 2023; Wang et al., 2016). However, two studies experienced sample attrition or data quality issues, potentially affecting integrity, and were assessed as high risk (Dudysová et al., 2024; Jespersen et al., 2019).

All studies fully reported predefined outcome measures (e.g., PSQI, ISI), with no evidence of selective reporting, resulting in a low-risk assessment (Chang et al., 2012; Dudysová et al., 2024; Harmat et al., 2008; Jespersen et al., 2019; Lai and Good, 2005; Tegeler et al., 2020; Tegeler et al., 2023; Wang et al., 2016).

**FIGURE 2:**
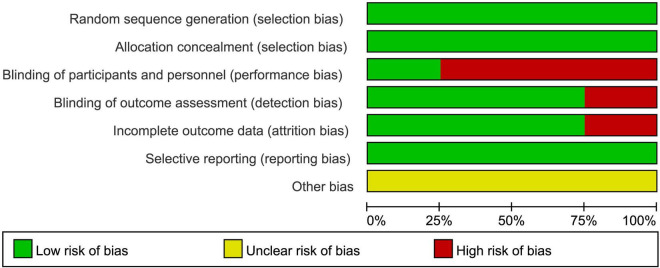
Risk of bias summary for the included randomized controlled trials.

For other biases, none of the studies provided detailed descriptions of potential sources of bias (e.g., environmental effects or participant expectations), making the risk unclear (Chang et al., 2012; Dudysová et al., 2024; Harmat et al., 2008; Jespersen et al., 2019; Lai and Good, 2005; Tegeler et al., 2020; Tegeler et al., 2023; Wang et al., 2016). The bias risk assessment chart is shown in Figure 2.

### 3.4 Meta-analysis of results

#### 3.4.1 The PSQI score

**FIGURE 3:**
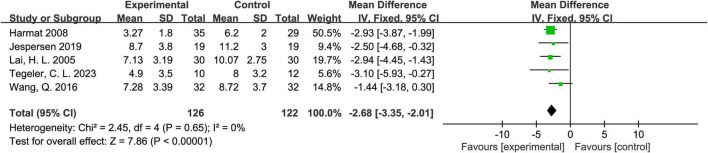
Forest plot of the effect of acoustic stimulation on the Pittsburgh Sleep Quality Index (PSQI).

Five studies reported PSQI scores (Harmat et al., 2008; Jespersen et al., 2019; Lai and Good, 2005; Tegeler et al., 2023; Wang et al., 2016). The heterogeneity test showed *P* = 0.65 and *I*^2^ = 0%, indicating very low heterogeneity among the studies. Therefore, a fixed-effects model was used. The meta-analysis results demonstrated that the experimental group performed significantly better than the control group, with an overall mean difference (MD) of -2.68 and a 95% CI of (-3.35, -2.01), showing statistical significance. The overall effect test (*Z* = 7.86, *P* < 0.00001) further confirmed the superior performance of the experimental group. Detailed results are shown in Figure 3.

#### 3.4.2 The TST score

**FIGURE 4:**
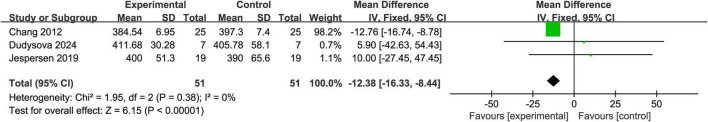
Forest plot of the effect of acoustic stimulation on total sleep time (TST).

Three studies reported TST (total sleep time) scores (Chang et al., 2012; Dudysová et al., 2024; Jespersen et al., 2019). The heterogeneity test yielded *P* = 0.38 and *I*^2^ = 0%, indicating very low heterogeneity among the studies; therefore, a fixed-effects model was applied. The meta-analysis revealed that the total sleep time in the experimental group was significantly shorter than that in the control group, with a mean difference (MD) of -12.38 and a 95% confidence interval (CI) of (-16.33, -8.44), indicating statistical significance (*Z* = 6.15, *P* < 0.00001). These results suggest that acoustic stimulation did not demonstrate a beneficial effect in prolonging total sleep time. Detailed results are shown in Figure 4.

#### 3.4.3 The SE score

**FIGURE 5:**
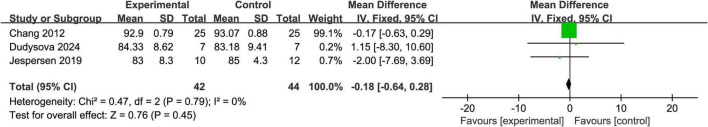
Forest plot of the effect of acoustic stimulation on sleep efficiency (SE).

Three studies reported SE (sleep efficiency) scores (Chang et al., 2012; Dudysová et al., 2024; Jespersen et al., 2019). The heterogeneity test showed *P* = 0.79 and *I*^2^ = 0%, indicating no significant heterogeneity among the studies; thus, a fixed-effects model was used. The meta-analysis showed an overall mean difference (MD) of -0.18 with a 95% CI of (-0.64, 0.28), which was not statistically significant (*Z* = 0.76, *P* = 0.45). These findings further suggest that there was no significant difference in sleep efficiency between the experimental and control groups. Detailed results are presented in Figure 5.

#### 3.4.4 The ISI score

**FIGURE 6:**
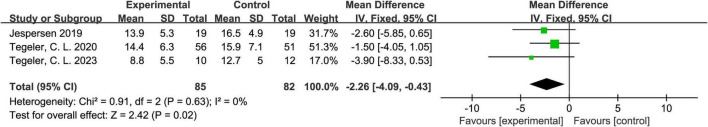
Forest plot of the effect of acoustic stimulation on the Insomnia Severity Index (ISI).

Three studies reported ISI (Insomnia Severity Index) scores (Jespersen et al., 2019; Tegeler et al., 2020; Tegeler et al., 2023). The heterogeneity test indicated *P* = 0.63 and *I*^2^ = 0%, suggesting no meaningful heterogeneity among the studies; hence, a fixed-effects model was appropriate. The meta-analysis showed that the experimental group outperformed the control group, with a mean difference (MD) of -2.26 and a 95% CI of (-4.09, -0.43), indicating statistical significance (*Z* = 2.42, *P* = 0.02). These results suggest that acoustic stimulation may be beneficial in alleviating insomnia symptoms. Detailed results are shown in Figure 6.

#### 3.4.5 Analysis of publication bias

**FIGURE 7:**
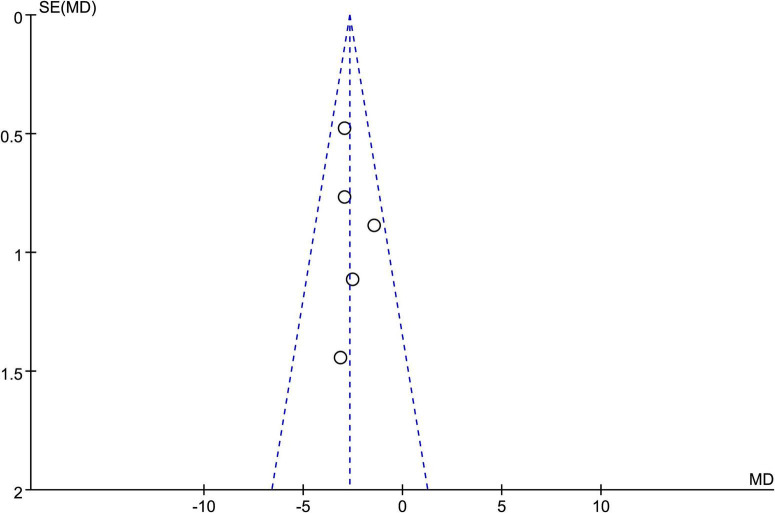
Funnel plot for assessing publication bias across included studies.

A funnel plot based on the PSQI scores of five studies showed that the data points were mostly distributed within the funnel-shaped area, with good symmetry on both sides, indicating a low likelihood of publication bias (Harmat et al., 2008; Jespersen et al., 2019; Lai and Good, 2005; Tegeler et al., 2023; Wang et al., 2016). However, the funnel plot provides only a visual assessment and cannot completely rule out other sources of bias. Therefore, we conducted a quantitative evaluation using statistical software (Stata 18.0). The Egger test yielded a result of *P* = 0.034 < 0.05, indicating the presence of some publication bias. Detailed results are shown in Figure 7.

## 4 Discussion

Insomnia is one of the most common sleep disorders, and its exact pathophysiological mechanisms remain unclear. However, research indicates that it is closely related to alterations in neural pathways, neuronal dysfunction, and disruptions in cellular homeostasis. Recent studies have proposed several potential mechanisms, including chronic neuroinflammation interfering with neural signaling and sleep-wake rhythms; abnormal activation of the hypothalamic-pituitary-adrenal axis leading to stress responses, such as elevated cortisol levels, which disrupt sleep regulation (Zhao and Luo, 2023); aberrant expression of vascular endothelial growth factor (VEGF) impairing cerebral blood flow regulation and neurovascular coupling (Wang et al., 2024); decreased levels of brain-derived neurotrophic factor (BDNF) reducing neuroplasticity and affecting sleep homeostasis; and dysfunction of monoaminergic neurotransmitters (e.g., serotonin and dopamine) causing imbalances in sleep-wake regulation. These findings provide critical insights into the pathophysiological processes of insomnia and offer a foundation for its clinical management (Shi et al., 2021).

Acoustic stimulation, as a non-pharmacological intervention, offers significant benefits for treating insomnia. Studies have shown that acoustic stimulation interventions can effectively improve sleep quality, shorten sleep onset latency, and enhance overall sleep efficiency. By regulating the autonomic nervous system, acoustic stimulation reduces sympathetic activity, lowers heart rate and blood pressure, and creates favorable conditions for sleep. Additionally, it influences neurochemical pathways by decreasing cortisol levels and promoting melatonin secretion, thereby facilitating sleep regulation. Acoustic stimulation also alleviates comorbid symptoms such as anxiety and depression, indirectly improving sleep quality. As a non-invasive and cost-effective approach with minimal risks, acoustic stimulation is easy to incorporate into daily life, making it an ideal alternative treatment for insomnia (Jespersen et al., 2022; Zhao et al., 2024).

Zeller et al. (2024) demonstrated that acoustic stimulation could enhance slow-wave sleep (SWS), a deep sleep state crucial for recovery and memory consolidation. The increase in slow-wave sleep is directly related to improved sleep quality and can help alleviate insomnia symptoms. Kanzler et al. (2023) also found that acoustic stimulation effectively reduces sleep onset latency, increases sleep duration, and decreases nocturnal awakenings, making it a viable intervention for insomnia. Marina Wunderlin et al. (2024) observed through polysomnography that participants receiving acoustic stimulation had a significantly higher proportion of slow-wave sleep compared to the control group, further confirming the effectiveness of acoustic stimulation in promoting deep sleep. Furthermore, Yan et al. (2024) studied the effects of bedtime music therapy on insomnia in college students, revealing that both classical and jazz music significantly improved insomnia severity and sleep quality, indicating that listening to music before sleep is effective in alleviating insomnia and enhancing sleep quality.

We considered analyzing the potential correlation between intervention duration and improvement in PSQI scores. However, due to the limited number of studies and inconsistent reporting of treatment duration, we were unable to conduct a statistically meaningful analysis. Nevertheless, previous research has suggested that longer durations of acoustic stimulation may yield greater improvements in sleep quality. Some studies reported a dose-response relationship between intervention length and PSQI reduction in clinical trials. This highlights the need for future studies to more systematically examine the impact of treatment duration on therapeutic outcomes.

This study conducted a meta-analysis of 8 studies and 419 cases, which indicated that acoustic stimulation is effective in treating insomnia. The meta-analysis of PSQI and ISI scores showed that acoustic stimulation significantly improved insomnia severity. Although no significant effect was observed on SE and TST acoustic stimulation effectively increased total sleep duration and alleviated insomnia symptoms.

### 4.1 Limitations

Although this meta-analysis suggests that acoustic stimulation may be effective in treating insomnia, several limitations should be noted. One concern is that the overall sample size of the included studies was relatively small, and only English-language publications were considered, which may restrict the generalizability of the findings. Moreover, the included studies lacked cross-cultural diversity and often exhibited imbalances in gender representation. Considerable variation was also observed in intervention parameters such as stimulation frequency, duration, and timing of application, contributing to methodological heterogeneity and limiting external validity. Future studies should adopt more standardized protocols and strive for demographically balanced and culturally diverse populations.

Another limitation relates to the classification of acoustic stimulation types. Although it has been suggested that different frequencies or sound forms (e.g., low-frequency noise vs. music) may have varying effects, most of the included studies did not clearly report stimulation frequency or spectral parameters. This precluded further subgroup analysis based on acoustic characteristics, which might otherwise have revealed important moderators of treatment efficacy.

The nature of the outcome measures also warrants consideration. While this review analyzed total sleep time (TST), sleep efficiency (SE), PSQI, and ISI, most of these measures were self-reported. Although objective data, such as those obtained from polysomnography (PSG), were extracted when available, the majority of included studies relied on subjective sleep assessments. This reliance introduces the potential for recall and reporting bias, which may affect the precision and reliability of the findings. Future studies should consider incorporating objective physiological indicators—such as salivary cortisol or serum BDNF—alongside self-reported scales to enable more comprehensive evaluations and facilitate mechanistic insights.

In addition, the pathophysiology of insomnia is multifactorial, involving variables such as age, sex, education, personality traits, and work-related stress. The included studies generally lacked detailed stratification by these baseline characteristics and did not clearly differentiate acoustic stimulation as a standalone intervention vs. combination with other therapies (Capezuti et al., 2022; Liu et al., 2023). These unaccounted factors may confound the interpretation of treatment effects. Furthermore, although functional neuroimaging techniques such as fMRI have been employed in some studies to investigate the neural mechanisms of auditory stimulation, none of the studies included in this meta-analysis used neuroimaging outcomes as either primary or secondary endpoints (Bliwise et al., 2014; Gathecha et al., 2016). As such, they did not meet our inclusion criteria. Nevertheless, future research integrating clinical outcomes with neuroimaging data may help clarify the underlying mechanisms and enhance mechanistic understanding of acoustic stimulation in insomnia treatment.

## 5 Conclusion

In conclusion, acoustic stimulation effectively improves the sleep status of insomnia patients and offers high safety and low cost, making it suitable for long-term treatment. As a non-invasive therapy, acoustic stimulation avoids the side effects associated with medication and reduces the strain on the body. It not only helps enhance sleep quality but also alleviates other symptoms related to insomnia, improving overall health. With advancements in technology, personalized treatment and smart monitoring of acoustic stimulation will further enhance its effectiveness, providing more feasible solutions for insomnia patients. Therefore, acoustic stimulation, as a simple, effective, and safe treatment, has broad clinical application prospects and deserves further research and promotion.

## Data Availability

The original contributions presented in the study are included in the article/supplementary material, further inquiries can be directed to the corresponding author.

## References

[B1] Bliwise D. Holm-Larsen T. Goble S. (2014). Increases in duration of first uninterrupted sleep period are associated with improvements in PSQI-measured sleep quality. *Sleep Med.* 15 1276–1278. 10.1016/j.sleep.2014.05.013 25172115 PMC4170006

[B2] Capezuti E. Pain K. Alamag E. Chen X. Philibert V. Krieger A. (2022). Systematic review: Auditory stimulation and sleep. *J. Clin. Sleep Med.* 18 1697–1709. 10.5664/jcsm.9860 34964434 PMC9163611

[B3] Chang E. Lai H. Chen P. Hsieh Y. Lee L. (2012). The effects of music on the sleep quality of adults with chronic insomnia using evidence from polysomnographic and self-reported analysis: A randomized control trial. *Int. J. Nurs. Stud.* 49 921–930. 10.1016/j.ijnurstu.2012.02.019 22494532

[B4] Dudysová D. Jankù K. Pioreckı M. Hantáková V. Orendáèová M. Piorecká V. (2024). Closed-loop auditory stimulation of slow-wave sleep in chronic insomnia: A pilot study. *J. Sleep Res.* 33:e14179. 10.1111/jsr.14179 38467353 PMC11597015

[B5] Gathecha E. Rios R. Buenaver L. Landis R. Howell E. Wright S. (2016). Pilot study aiming to support sleep quality and duration during hospitalizations. *J. Hosp. Med.* 11 467–472. 10.1002/jhm.2578 26970217

[B6] Harmat L. Takács J. Bódizs R. (2008). Music improves sleep quality in students. *J. Adv. Nurs.* 62 327–335. 10.1111/j.1365-2648.2008.04602.x 18426457

[B7] Javaheri S. Redline S. (2017). Insomnia and risk of cardiovascular disease. *Chest* 152 435–444. 10.1016/j.chest.2017.01.026 28153671 PMC5577359

[B8] Jespersen K. Otto M. Kringelbach M. Van Someren E. Vuust P. A. (2019). randomized controlled trial of bedtime music for insomnia disorder. *J. Sleep Res.* 28:e12817. 10.1111/jsr.12817 30676671

[B9] Jespersen K. Pando-Naude V. Koenig J. Jennum P. Vuust P. (2022). Listening to music for insomnia in adults. *Cochrane Database Syst. Rev.* 8:CD010459. 10.1002/14651858.CD010459.pub3 36000763 PMC9400393

[B10] Kanzler S. Cidral-Filho F. Kuerten B. Prediger R. (2023). Effects of acoustic neurostimulation in healthy adults on symptoms of depression, anxiety, stress and sleep quality: A randomized clinical study. *Explor. Neuroprot. Ther.* 3 481–496. 10.37349/ent.2024.00086 39280247

[B11] Lai H. Good M. (2005). Music improves sleep quality in older adults. *J. Adv. Nurs.* 49 234–244. 10.1111/j.1365-2648.2004.03281.x 15660547

[B12] Liu S. Zhang S. Guo M. Lei Q. He L. Li Z. (2023). Acoustic stimulation during sleep improves cognition and ameliorates Alzheimer’s disease pathology in APP/PS1 mice. *Exp. Gerontol.* 182:112299. 10.1016/j.exger.2023.112299 37776987

[B13] Morin C. M. Benca R. (2012). Chronic insomnia. *Lancet* 379 1129–1141. 10.1016/S0140-6736(11)60750-2 22265700

[B14] Morin C. M. Buysse D. J. (2024). Management of insomnia. *N. Engl. J. Med.* 391 247–258. 10.1056/NEJMcp2305655 39018534

[B15] Shi Y. Wang Z. X. Guo H. Sun P. Y. Feng Y. C. (2021). Research progress on the pathogenesis of insomnia and treatment strategies in Traditional Chinese and Western Medicine. *Dangdai Yiyao Luncong* 19, 16–18. 10.3969/j.issn.2095-7629.2021.22.008

[B16] Tegeler C. Munger Clary H. Shaltout H. Simpson S. Gerdes L. Tegeler C. (2023). Cereset research standard operating procedures for insomnia: A randomized, controlled clinical trial. *Glob. Adv. Integr. Med. Health* 12:27536130221147475. 10.1177/27536130221147475 36816469 PMC9933987

[B17] Tegeler C. Shaltout H. Lee S. Simpson S. Gerdes L. Tegeler C. (2020). High-resolution, relational, resonance-based, electroencephalic mirroring (HIRREM) improves symptoms and autonomic function for insomnia: A randomized, placebo-controlled clinical trial. *Brain Behav.* 10:e01826. 10.1002/brb3.1826 32940419 PMC7667311

[B18] Wang Q. Chair S. Wong E. Li X. (2016). The effects of music intervention on sleep quality in community-dwelling elderly. *J. Altern. Complement. Med.* 22 576–584. 10.1089/acm.2015.0304 27223689

[B19] Wang Y. Ma W. Kong L. Zhang H. Yuan P. Qu W. (2024). Ambient chemical and physical approaches for the modulation of sleep and wakefulness. *Sleep Med. Rev.* 79 102015. 10.1016/j.smrv.2024.102015 39447526

[B20] Wunderlin M. Zeller C. J. Wicki K. (2024). Acoustic stimulation during slow wave sleep shows delayed effects on memory performance in older adults. *Front. Sleep* 2:1294957. 10.3389/frsle.2023.1294957 PMC12713973 41426477

[B21] Yan D. Wu Y. Luo R. Yang J. (2024). Bedtime music therapy for college students with insomnia: A randomized assessor-blinded controlled trial. *Sleep Med.* 121 326–335. 10.1016/j.sleep.2024.07.018 39053128

[B22] Zeller C. Wunderlin M. Wicki K. Teunissen C. Nissen C. Züst M. (2024). Multi-night acoustic stimulation is associated with better sleep, amyloid dynamics, and memory in older adults with cognitive impairment. *Geroscience* 46 6157–6172. 10.1007/s11357-024-01195-z 38744792 PMC11493878

[B23] Zhao N. Lund H. Jespersen K. V. (2024). A systematic review and meta-analysis of music interventions to improve sleep in adults with mental health problems. *Eur. Psychiatry* 67:e62. 10.1192/j.eurpsy.2024.1773 39373544 PMC11536203

[B24] Zhao Y. H. Luo X. (2023). Research progress on the epidemiology and pathogenesis of insomnia. *Chin. J. Clin. Phys.* 51, 1397–1401. 10.3969/j.issn.2095-8552.2023.12.004

